# Efficiency and mechanisms of rhodamine B degradation in Fenton-like systems based on zero-valent iron

**DOI:** 10.1039/d0ra03125a

**Published:** 2020-08-03

**Authors:** Liping Liang, Liubiao Cheng, Yuting Zhang, Qian Wang, Qian Wu, Yuanyuan Xue, Xu Meng

**Affiliations:** School of Civil Engineering, Shaoxing University Shaoxing 312000 P. R. China liangliping0702@163.com; College of Life Science, Shaoxing University Shaoxing 312000 P. R. China; College of Textile and Garment, Shaoxing University Shaoxing 312000 P. R. China mengxu0@163.com; Key Laboratory of Clean Dyeing and Finishing Technology of Zhejiang Province, Shaoxing University Shaoxing 312000 China

## Abstract

Based on the Fe^0^/H_2_O_2_ heterogeneous Fenton system, the degradation of rhodamine B (RhB, an organic dye pollutant) was researched in this paper. The effects of initial pH value, concentration of H_2_O_2_, dosage of zero-valent iron (ZVI), and initial RhB concentration on RhB degradation by Fe^0^/H_2_O_2_ were studied. The results showed that when the initial pH = 4, dosage of ZVI was 9 mM, and concentrations of H_2_O_2_ and RhB were 8 mM and 0.1 mM, respectively, the color of RhB could be completely faded within 30 min, and the total organic carbon (TOC) removal percentage was about 63% after 120 min. The dissolved oxygen (DO) content and oxidation–reduction potential (ORP) were monitored during the reaction. Quenching experiments with methanol confirmed that the degradation of the dye was mainly due to oxidation by the ˙OH radical. Besides, the results from UV-Vis spectroscopy showed that the degradation of RhB was mainly due to the destruction of the conjugated oxygen hetero-anthracene in the RhB molecule. The solid-phase characterization of the ZVI samples after reaction confirmed that the original regular and slippery ZVI samples finally were corroded into rough and irregular lepidocrocite and magnetite. Two possible competitive reaction pathways for the degradation of RhB by Fe^0^/H_2_O_2_ were proposed by GC-MS analysis, which were attributed to the dissociation of ethyl radicals and the degradation of chromophore radicals.

## Abstract 2

Based on the Fe^0^/H_2_O_2_ heterogeneous Fenton system, the degradation of rhodamine B (RhB, an organic dye pollutant) was researched in this paper.

## Graphical abstract

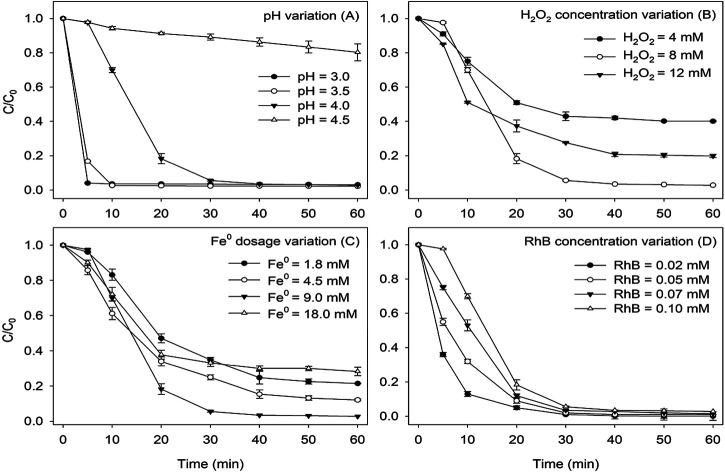

## 1. Introduction

In 2015, Chinese industry wastewater treatment capacity reached 20.0 billion tons, and the discharge wastewater from the manufacture of textiles, clothing, and apparel was estimated to be 2.0 billion tons.^1^ Thus, dye wastewater from the textile, dyeing, and printing industries has been a serious environmental problem. Generally, most dyestuffs present in dye and textile wastewater possess complicated chemical structures, such as hydroxytriarylmethanes, xanthene, and aminotriarylmethanes, which are difficult to destroy by biological or photolytic processes. Rhodamine B (RhB), as an important aminoxanthene dye, is widely used as a textile, biological and fluorescent stain, in the colored glass industry, and in fireworks.^2,3^ RhB is also used as a food additive in some developing countries, such as India,^4^ Vietnam,^5^ and Argentina.^6^ However, RhB has been listed as a carcinogenic chemical (Group 3) by the IARC (International Agency for Research on Cancer) since 1987.^7^ Therefore, many developed countries and regions such as Japan, the EU, and the United States have forbidden the use of RhB.^8^ Besides, RhB has toxic effects on animals and humans, inducing teratogenicity, carcinogenicity and mutagenicity.^9^ RhB is also frequently present in dye wastewater due to the wide application of RhB in the textile industry. RhB in wastewater easily accumulates, but it can be effectively degraded in an oxidation system.^10,11^ Therefore, it is very important to find a suitable oxidation system to treat wastewater.

Traditional Fenton reaction^12^ is considered as a good strategy to treat dye wastewater. Its essence is that H_2_O_2_ can produce hydroxyl radicals (˙OH) with high reaction activity under the catalytic action of Fe^2+^, and most organic matter can be degraded by ˙OH. However, the traditional Fenton reaction is limited by the addition of a high concentration of H_2_O_2_, the narrow effective pH range, and the production of iron-containing sludge.^13,14^ Besides, some ligands, such as tetraamido macrocyclic ligand (TAML),^15^ were coupled with Fe(iii) to catalyze H_2_O_2_ to degrade dyes under suitable pH conditions. But the addition of organic ligands is high cost and might induce secondary pollution in the water environment. As a kind of material that is easy to obtain, low cost, and friendly towards the environment, ZVI can replace the addition of ferrous salt in advanced oxidation processes (AOPs). Zero-valent iron (ZVI), as a green reductant (*E*_0_ = −0.44 V for Fe^2+^/Fe^0^),^16^ has exhibited good performance with various organic and inorganic compounds, including oxygen heteroanthracene dye removal from the aqueous phase.^17^ However, the microscale ZVI powder always possessed a lower reactivity, especially under neutral or alkaline conditions.^18,19^ Besides, ZVI always reduced RhB, making it difficult to destroy the chemical structure of RhB. So, the addition of some oxidants such as H_2_O_2_ into ZVI systems could establish a heterogeneous Fenton-like system which enhances the removal of RhB.

Güçlü *et al.*^20^ attempted to use ZVI instead of ferrous salt as a potent source of divalent iron in the Fenton reaction system. In the presence of H_2_O_2_, O_2_ or natural organic compounds, various redox reactions were driven.^21–24^ The main principle of Fe^0^/H_2_O_2_ systems is that ZVI is oxidized to Fe^2+^*via* two-electron transfer from the surface of the particle to H_2_O_2_.^25^ The reaction between Fe^2+^ and H_2_O_2_ can generate ˙OH and Fe^3+^.^26^ Then, Fe^3+^ could further interact with the surface of ZVI, and is reduced to Fe^2+^.^27^ Moreover, the heterogeneous Fenton based on ZVI is usually used at a low pH and a high concentration of H_2_O_2_.^28,29^ Cabrera *et al.*^30^ found the herbicide diuron was 99% degraded in Fe^0^/H_2_O_2_ system after 10 minutes. Although Fe^0^/H_2_O_2_ systems have been applied to degrade various organics, including dyes, the degradation of RhB by the Fe^0^/H_2_O_2_ system is rarely studied. Therefore, this study will explore the degradation of RhB by the Fe^0^/H_2_O_2_ system.

In this paper, RhB was used as a model pollutant to investigate the factors affecting the reaction rate and the effects of Fenton reaction catalysis by ZVI under a low concentration of H_2_O_2_. The purpose of the present study is to determine the effects of various parameters, including temperature, initial pH, ZVI dosage, and hydrogen peroxide concentration in Fe^0^/H_2_O_2_ systems; clarify the mechanism of RhB removal by Fe^0^/H_2_O_2_; and explore the degradation pathway of RhB in the Fe^0^/H_2_O_2_ process.

## 2. Materials and methods

### 2.1 Materials

All chemicals were analytical grade and used in the required manner. The 30% hydrogen peroxide was purchased from Shanghai Lingfeng Chemical Reagent Co., Ltd., and RhB was produced by Jinan Xucheng Dye Chemical Co., Ltd. ZVI samples were obtained from Shanghai Haotian Nano Technology Co., Ltd.

### 2.2 Experimental method

RhB stock solution (1 g L^−1^) was stored in a brown reagent bottle to prevent dye deterioration. A wide-mouth bottle filled with the 500 mL solution containing RhB was placed in the sink. The water in the sink was circulated by a water circulator, which was set at 25 °C. An electric mixer was employed to mix the solution at 400 rpm. Then, the experiment was installed by adding hydrogen peroxide and ZVI samples simultaneously. At 0, 5, 10, 20, 30, 40, 50, and 60 min, samples were taken with a disposable needle and passed through a membrane filter with pore size of 0.45 μm to remove suspended solids from the water. A few drops of sodium thiosulfate quencher were added to the sample to stop the reaction. All experiments were performed in three groups under the given conditions; all points in the graph represent the average, and error bars indicate standard deviation.

### 2.3 Chemical analysis

The concentration of RhB was measured by UV-vis spectrophotometer (Cary 50, Agilent Corporation) at 554 nm. Oxidation–reduction potential (ORP) and dissolved oxygen (DO) content of the sample were monitored by ORP and DO sensors connected to the PHS-3C pH meter (FE20, Mettler-Toledo Instruments Co., Ltd.). Selected samples at fixed time intervals were scanned by UV-Vis at 250–650 nm to monitor the variation of intermediate products during the degradation of RhB. The reacted solution was lyophilized, and the dried solid sample was stored in a vacuum bag. The crystal morphology of iron oxides and iron hydroxides in solid products at different pH levels was analyzed by XRD (Empyrean, Dutch Panalytical Co., Ltd.). The morphology of the solid product after the reaction at different pH levels was observed by SEM (JSM-6360LV, Japan Electronics Co., Ltd.).

### 2.4 GC-MS pretreatment method and conditions

100 mL of water sample was filtered through a 0.45 μm filter, then 25 mL dichloromethane was added for mixed oscillatory static delamination, and the extraction was repeated three times. The organic phase was transferred to a vacuum rotary evaporator, then concentrated to 3–5 mL at 40 °C, and blown off to 1 mL with nitrogen. The gas chromatograph (Agilent 7890A) was equipped with a HP-17 ms quartz capillary column (30 m × 0.25 mm, 0.25 μm), which was interfaced directly to the mass spectrometer (5975A inert XL MSD). The GC column was operated in a temperature-programmed mode with an initial temperature of 40 °C, maintained for 2 min, then the temperature was increased to 160 °C at a speed of 10 °C min^−1^, kept for 2 min, and heated to 250 °C at 20 °C min^−1^. The quality scanning range was 45–600 *m*/*z*, electron bombardment source EI, electronic energy 70 eV, electron multiplier voltage 2400 V, and electron source temperature 250 °C. The product analysis was consulted against the NIST08 mass spectral library database.

## 3. Results and discussion

### 3.1 Effects of different reaction parameters

#### 3.1.1 Effects of initial pH value

**Fig. 1:**
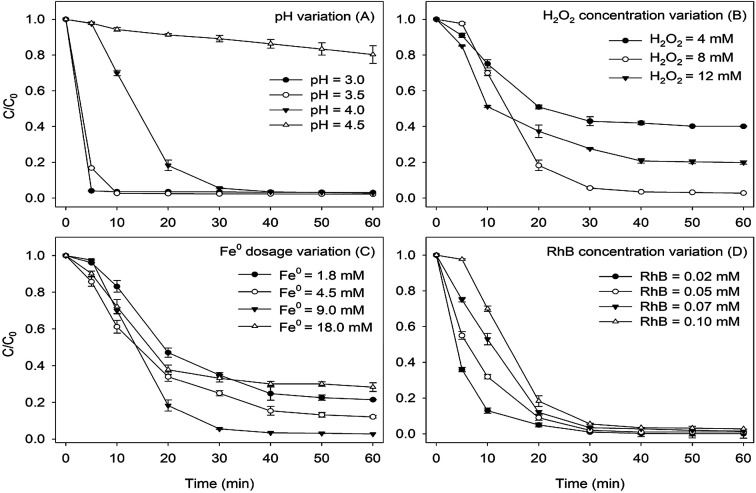
Effect of operating conditions (pH, H_2_O_2_ concentration, Fe^0^ dosage, and RhB concentration) on the removal of RhB by Fenton-like system [(A) [Fe^0^] = 9 mM, [H_2_O_2_] = 8 mM, [RhB] = 0.1 mM, and *T* = 25 °C; (B) [Fe^0^] = 9 mM, pH = 4.0, [RhB] = 0.1 mM, and *T* = 25 °C; (C) pH = 4.0, [H_2_O_2_] = 8 mM, [RhB] = 0.1 mM, and *T* = 25 °C; (D) [Fe^0^] = 9 mM, pH = 4.0, [H_2_O_2_] = 8 mM, and *T* = 25 °C].

The effects of pH on RhB removal by Fe^0^/H_2_O_2_ were studied in the pH range of 3.0–4.5. As shown in Fig. 1A, the Fenton-like reaction is significantly affected by pH. Under the same conditions, the degradation rate of RhB was seriously inhibited with the increase of pH. When pH was less than 4, the RhB could almost completely be removed in 30 min. However, the removal percentage of RhB could only reach 20% after 60 min reaction at pH 4.5. Thus, RhB removal by the heterogeneous Fenton reaction was obviously limited by the range of pH.^31^ With the increase of H^+^ concentration, the corrosion of ZVI was accelerated, which could be conducive to providing sufficient Fe^2+^. The reaction between Fe^2+^ and H_2_O_2_ also was enhanced with the decrease of pH, and the lower pH enhanced the generation of ˙OH (eqn (1)). Moreover, the oxidation potential of ˙OH increased with the decrease of pH, thus giving it a stronger oxidation ability.^32^

1 Fe^2+^ + H_2_O_2_ → Fe^3+^ + OH^−^ + ˙OH

#### 3.1.2 Effects of initial H_2_O_2_ concentration

The concentration of H_2_O_2_ is also a key parameter of Fenton-like reaction. Compared with the traditional Fenton reaction, one of the advantages in Fenton-like reaction systems is the low concentration of H_2_O_2_.^33^ The effects of initial H_2_O_2_ concentration on the degradation of RhB by Fe^0^/H_2_O_2_ are illustrated in Fig. 1B. With the increase of H_2_O_2_ concentration from 4 mM to 8 mM, the degradation rate of RhB increased. At 60 min, the degradation percentage of RhB reached 98% at the concentration of 8 mM H_2_O_2_. By properly increasing the concentration of H_2_O_2_, more hydroxyl radicals could be produced, which could promote the degradation percentage of RhB. However, when the concentration of H_2_O_2_ increased to 12 mM, the degradation percentage of RhB decreased, which was due to the fact that the high-concentration H_2_O_2_ becomes a hydroxyl-shielding agent and forms ˙HO_2_ with less oxidation ability than ˙OH, as revealed by eqn (2).^34^ In addition, it could continue to react with hydroxyl radical to release O_2_ (eqn (3)),^34^ which consumes some hydroxyl radical and hinders the mineralization of RhB.

2 H_2_O_2_ + ˙OH → H_2_O + ˙HO_2_

3 ˙HO_2_ + ˙OH → H_2_O + O_2_

#### 3.1.3 Effects of initial ZVI concentration

ZVI was the main source of Fe^2+^ in the Fenton-like system. The effects of ZVI dosage on the removal efficiency of RhB were investigated experimentally. As shown in Fig. 1C, when Fe^0^ = 1.8 mM, the removal percentage reached 79% in 60 min. With the increase of ZVI concentration from 1.8 mM to 9 mM, the removal percentage was gradually increased. The active sites on ZVI surface were increased with the increasing concentration of ZVI, and the generation of hydroxyl radicals was promoted. However, when the dosage of ZVI reached 18 mM, the degradation percentage of RhB decreased. It is possible that excess ZVI could react with H_2_O_2_ to form Fe^2+^, which could be represented by eqn (4). In addition, excess Fe^2+^ would react with ˙OH to generate Fe^3+^ and OH^−^ (eqn (5)). The hydroxyl radical was consumed in this way, thus reducing the dye degradation efficiency.^35,36^

4 Fe^0^ + H_2_O_2_ → Fe^2+^ + 2OH^−^

5 Fe^2+^ + ˙OH → Fe^3+^ + OH^−^

#### 3.1.4 Effects of initial RhB concentration

The oxidation rate of the Fenton-like reaction is also related to the concentration of target organics. As illustrated in Fig. 1D, the effects of different dye concentrations on RhB removal by Fe^0^/H_2_O_2_ are investigated. The results showed that the removal percentage of RhB could reach 95% in 20 min at a low RhB concentration of 0.02 mM. Increasing RhB could occupy more active sites on the ZVI surface, which was not conducive to the reaction between ZVI reactive sites and H_2_O_2_ and led to a decrease in the percentage of hydroxyl radicals produced.

### 3.2 Variation of ORP and DO during RhB degradation

**Fig. 2:**
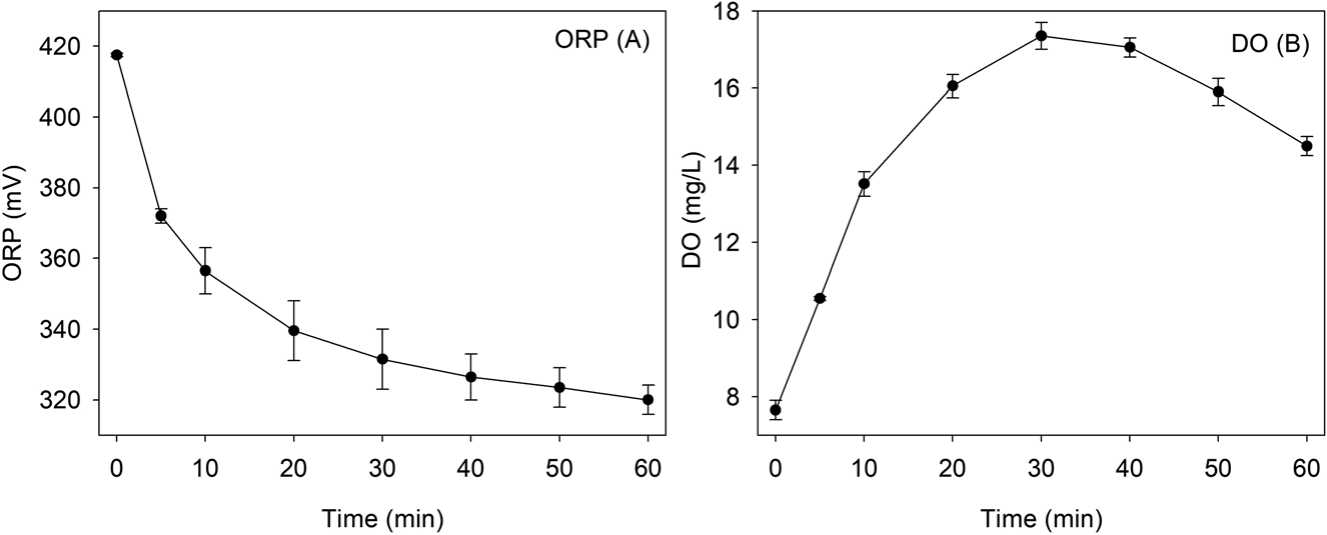
The change of ORP (A) and DO (B) during the removal of RhB using the Fenton-like system [pH = 4.0, [RhB] = 0.1 mM, [Fe^0^] = 9 mM, [H_2_O_2_] = 8 mM, and *T* = 25 °C].

The ORP was a mixed potential composed of the weighted sum of Nernstian terms for each of the redox couples that were present at the electrode surface.^37^ During the degradation of RhB by Fe^0^/H_2_O_2_, the redox couples contributing to the ORP value mainly include H_2_/H^+^, O_2_/OH^−^ and Fe^0^/Fe^2+^. The variation of DO and ORP values during the reaction are monitored under the optimal conditions of pH_ini_ = 4.0, H_2_O_2_ = 8 mM, RhB = 0.1 mM, Fe^0^ = 9 mM, and *T* = 25 °C, as illustrated in Fig. 2A and B. ORP value is positive during the whole reaction. Thus, the whole process was mainly an oxidation reaction. DO value gradually increased in the first 30 min to 17.4 mg L^−1^, then decreased to 14.5 mg L^−1^ at 60 min. The decomposition of hydrogen peroxide could produce oxygen, while the oxidation reactions of ZVI and RhB both consumed oxygen. In the first 30 min of reaction, the production of oxygen was higher than the consumption. Then, oxygen was consumed over the whole reaction process for the degradation of RhB and the corrosion of ZVI.

### 3.3 Quenching experiments

**Fig. 3:**
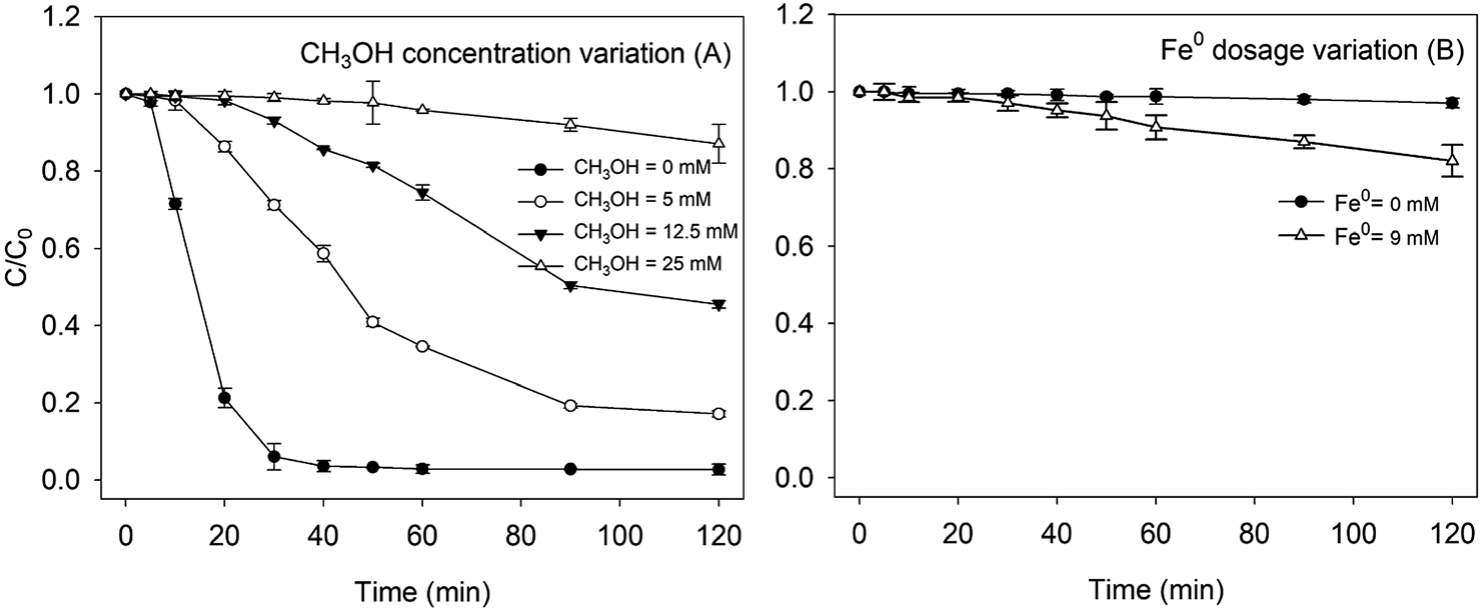
Effect of methyl alcohol on the removal of RhB by the Fenton-like system [(A) pH = 4.0, [RhB] = 0.1 mM, [Fe^0^] = 9 mM, [H_2_O_2_] = 8 mM, and *T* = 25 °C; (B) pH = 4.0, [RhB] = 0.1 mM, [H_2_O_2_] = 8 mM, and *T* = 25 °C].

In order to study the mechanisms of RhB degradation in a heterogeneous Fenton system, quenching experiments were conducted with methanol. As shown in Fig. 3, with the increase of methanol concentration, the removal of RhB by Fe^0^/H_2_O_2_ was seriously inhibited. When the amount of methanol added was more than 25 mM, the reaction hardly proceeded within 120 min, which indicated that the hydroxyl radical was the main reactive oxidative species for oxidizing RhB in the Fe^0^/H_2_O_2_ system. The degradation percentage of RhB was only about 15% at 120 min in the presence of 25 mM methanol. As shown in Fig. 3B, the removal percentage of RhB by ZVI alone after 120 min reached 18%. The slight removal of RhB indicated that RhB may be removed in other ways besides ˙OH oxidation, which was verified by a separate ZVI control experiment.^38^

**Fig. 4:**
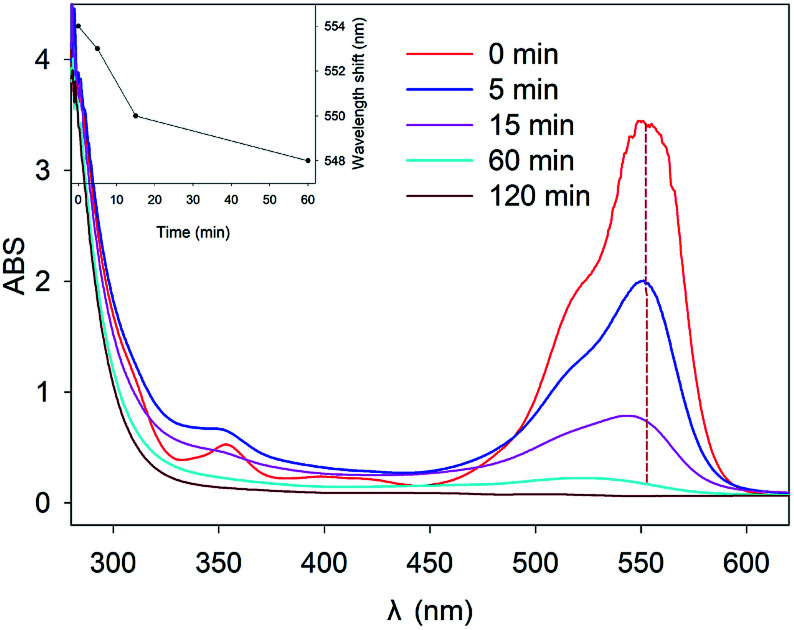
UV-Vis scanning spectra of RhB removal by Fenton-like system (Inset: the variation of the absorbance of RhB at 554 nm with time) [pH = 4.0, [Fe^0^] = 9 mM, [H_2_O_2_] = 8 mM, [RhB] = 0.1 mM, and *T* = 25 °C].

Fig. 4 illustrates the changes in UV-Vis spectra during RhB removal by Fe^0^/H_2_O_2_. The characteristic absorption peak of RhB at 554 nm decreased rapidly with reaction time. After 120 min reaction, the main chromophore of RhB disappeared, and the reaction solution quickly became colorless. But a weak blue shift phenomenon was observed in the spectrum, with its shift number as Δ*λ* = 6 nm (548–554 nm), which was consistent with the results reported by other researchers.^39^ This was due to the formation of *N*-de-ethylated intermediates, which indicated that the energy required for electron transition might have increased and the molecular structure of RhB changed during the degradation process.^40–42^

### 3.4 XRD and SEM solid-phase characterization

**Fig. 5:**
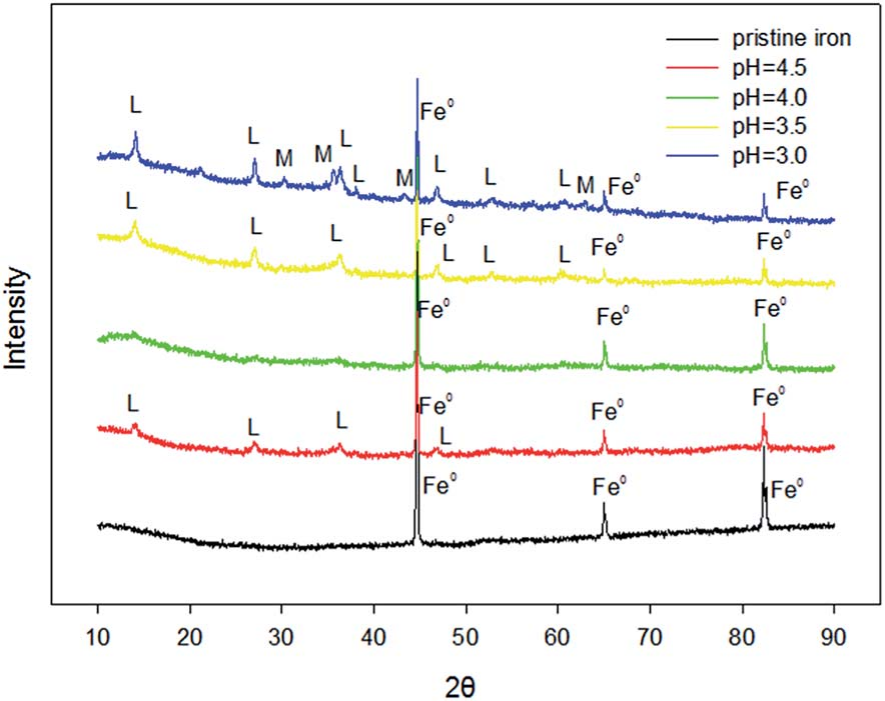
XRD patterns for different pH levels in the removal of RhB by the Fenton-like system [[Fe^0^] = 9 mM, [H_2_O_2_] = 8 mM, [RhB] = 0.1 mM, and *T* = 25 °C].

In order to study the corrosion mechanisms of ZVI during RhB removal by Fe^0^/H_2_O_2_, the corrosion products and the morphology of ZVI after the reaction at pH_ini_ = 3.0, 3.5, 4.0, and 4.5 were studied. As shown in Fig. 5, the initial ZVI sample has diffraction peaks at 2*θ* = 45°, 66°, and 83°. At different pH values, the peak intensity of ZVI samples after reaction at the same position decreased with the decrease of pH. According to the principle of quantitative phase analysis, the intensity of the diffraction peak of the phase to be measured is proportional to its content. So, more residual reactive sites of ZVI samples were detected with the increase of pH. Besides, the diffraction spectrum of the corrosion product also had characteristic peaks at 2*θ* = 13°, 28°, and 37°, characteristic of a lepidocrocite. The intensity of the peak increased with the decrease of pH. The results showed that ZVI was more easily corroded to lepidocrocite in the presence of H_2_O_2_ at a lower pH. Magnetite also was detected in the corrosion product of ZVI samples at pH_ini_ = 3.

**Fig. 6:**
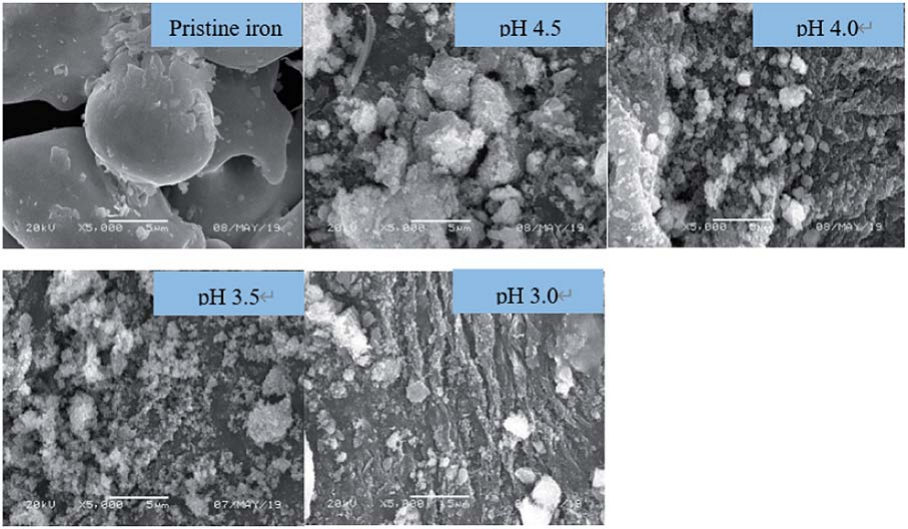
SEM images of ZVI samples after Fenton-like reaction at different pH levels [[Fe^0^] = 9 mM, [H_2_O_2_] = 8 mM, [RhB] = 0.1 mM, and *T* = 25 °C].

The morphologies of ZVI samples after reaction are shown in Fig. 6. With the increase of pH, the surface morphology of ZVI samples changes from regular and smooth particles to rough and irregular objects, and finally completely corrodes. At different pH_ini_ values of 3.0, 3.5, 4.0, and 4.5, the aging iron was mainly irregular flakes or needle shaped after 1 h, which might be ascribed to the iron oxide or iron hydroxide formed on the surface of ZVI. The change of corrosion morphology was consistent with the formation of magnetite and lepidocrocite in XRD.

### 3.5 Possible degradation products of RhB degradation by Fe^0^/H_2_O_2_

**Fig. 7:**
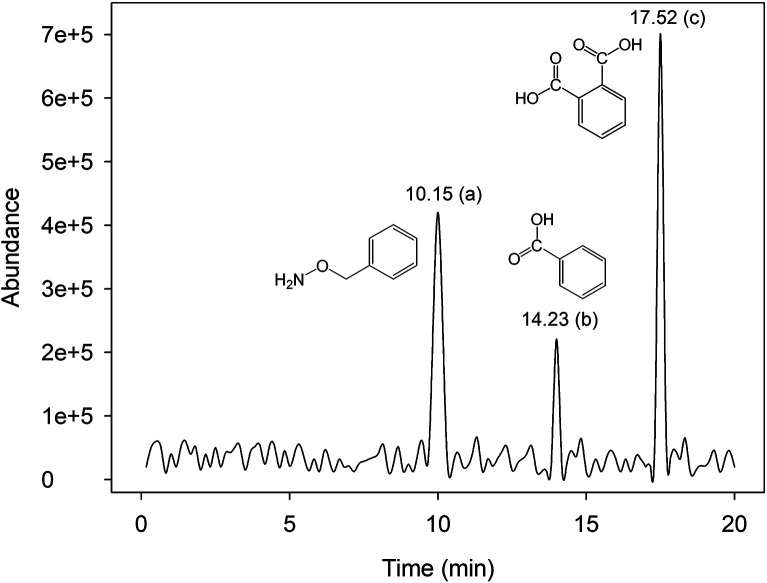
GC chromatograph of the derivatization products with the Fenton-like system [pH = 4.0, [Fe^0^] = 9 mM, [H_2_O_2_] = 8 mM, [RhB] = 0.1 mM, and *T* = 25 °C].

The analysis of intermediate products was done by GC-MS technique to understand RhB degradation mechanisms in Fe^0^/H_2_O_2_ systems, and the main degradation pathways of RhB were inferred. Fig. 7 shows the chromatograph of intermediate products obtained from GC-MS. Three main intermediate products were determined using the mass spectrometry database, and Table 1 shows specific information for each product.

**Table 1:** Identification of the intermediates of RhB during the degradation by GC-MS

Product	*R*_t_ (min)	MW	Formula	Name
a	10.05	123	C_7_H_9_NO	Benzyloxyamine
b	14.23	122	C_7_H_6_O_2_	Benzoic acid
c	17.52	166	C_8_H_6_O_4_	Phthalic acid

In Fe^0^/H_2_O_2_ systems, hydroxyl radicals could directly attack the central carbon of RhB, which breaks the RhB molecules and decolorizes the solution rapidly. During this reaction, benzene-ring substances such as benzoic acid, phthalic acid, and benzyloxyamine are mainly produced.^42^

**Fig. 8:**
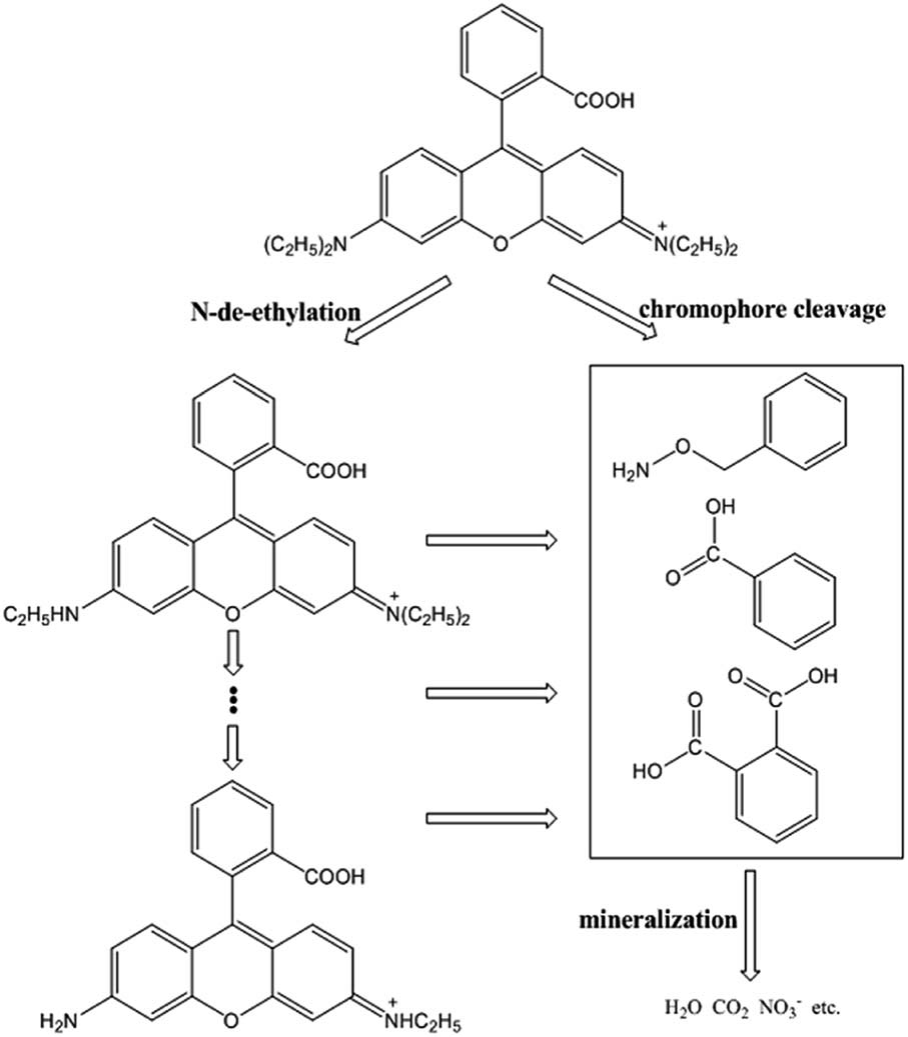
Degradation pathway of RhB by Fenton-like system.

**Fig. 9:**
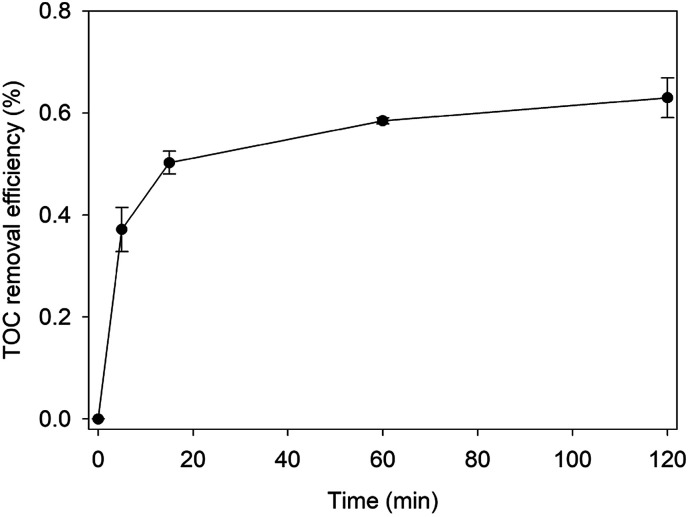
TOC removal percent of RhB by Fenton-like system [pH = 4.0, [RhB] = 0.1 mM, [Fe^0^] = 9 mM, [H_2_O_2_] = 8 mM, and *T* = 25 °C].

Based on the above experimental results and previous studies, the pathway of possible degradation of RhB in Fe^0^/H_2_O_2_ systems was proposed, as demonstrated in Fig. 8. The first step involves *N*-de-ethylation, which is a step-by-step process.^43^ Highly active hydroxyl radicals not only could attack the structural centres, resulting in RhB decomposition, but also attack the *N*-ethyl on the N site to break it. This was because the p-type electron orbital of nitrogen combined with the p-type electron orbital of the benzene ring to form a conjugated system. Some studies confirmed the existence of *N*-de-ethylation by LC-ESI-MS/MS method, but only to a certain extent in the observable range.^44^*N*-Ethyl is a color-assisting group with an auxiliary color effect.^45^ However, the decoloration of RhB was mainly the result of cleavage of the chromophoric conjugated group structure. At the same time, the destruction of the dye molecule conjugate system occurs, and the hydroxyl radical can directly attack the RhB center carbon, so the dye rapidly decolorizes to some extent. The *N*-ethyl group was affected by the conjugated system and was easily removed by the hydroxyl radical attack.^46^ So *N*-de-ethylated intermediates are also attacked by hydroxyl radicals to form some primary oxidation products, such as benzyloxyamine, benzoic acid and phthalic acid.^47^ TOC is an important index to judge the degree of BPA mineralization directly. The removal kinetics of TOC during the degradation of RhB is shown in Fig. 9. The TOC decreased with reaction time, and the TOC removal percent could reach 63% after 120 min. Hereafter, most substances with benzene ring are further degraded into smaller compounds, and small molecular compounds are eventually mineralized into CO_2_, H_2_O, NH_4_^+^, and so on. Therefore, two competitive processes of aryl chromophore degradation and *N*-ethyl dissociation occurred simultaneously during the reaction.

The heterogeneous Fenton-like reaction may be applied as an attractive alternative for removing other organic compounds in wastewater. More work is needed to handle the recovery of catalyst for reuse and expanding the pH range of the reaction, which may form the basis of an effective application for environmental treatment.

## 4. Conclusions

The heterogeneous Fenton system is composed of ZVI and H_2_O_2_, and the oxygen anthracene dye RhB could be effectively degraded by Fe^0^/H_2_O_2_. The optimal reaction conditions include a dye solution pH_ini_ of 4.0, H_2_O_2_ concentration of 8 mM, and Fe^0^ concentration of 9 mM; under these conditions, the degradation of 0.1 mM RhB solution reached 98% within 60 min. About 63% TOC could be removed after 120 min. The strong oxidation ability of Fe^0^/H_2_O_2_ systems is mainly due to the action of hydroxyl radicals, which exhibited a strong ability to degrade RhB. The ZVI samples were severely corroded after the reaction, and the surface was covered with iron oxides and hydroxides mainly composed of magnetite and lepidocrocite. There are two competitive pathways to degrade RhB, one is an *N*-de-ethylation process, and the other is cracking of the chromophore. However, the destruction of the chromophore structure is mainly the first pathway, producing primary oxidation products such as benzyloxyamine, benzoic acid, and phthalic acid. After a series of oxidations, the intermediates could finally be mineralized into CO_2_, H_2_O, NH_4_^+^, and so on.

## Conflicts of interest

There are no conflicts to declare.

## Supplementary Material
